# Exploratory Covalent Docking of Michael-Acceptor Natural Products at Reactive Cysteines in Cancer Tyrosine Kinases

**DOI:** 10.3390/ijms262311390

**Published:** 2025-11-25

**Authors:** Fernando Lobo, José Manuel Pérez de la Lastra, Celia María Curieses, Elena Bustamante-Munguira, Celia Andrés Juan, Eduardo Pérez-Lebeña

**Affiliations:** 1 Institute of Natural Products and Agrobiology, CSIC-Spanish Research Council, Avda. Astrofísico Fco. Sánchez, 3, 38206 La Laguna, Spain; ebustamante@saludcastillayleon.es; 2 Hospital Clínico Universitario de Valladolid, Avenida de Ramón y Cajal, 3, 47003 Valladolid, Spain; cmcuriesesa@saludcastillayleon.es; 3 Department of Organic Chemistry, Cinquima Institute, Faculty of Sciences, Valladolid University, Paseo de Belén, 7, 47011 Valladolid, Spain; celia.andres.juan@uva.es; 4 Sistemas de Biotecnología y Recursos Naturales, 47625 Valladolid, Spain; info@glize.eu

**Keywords:** cancer, tyrosine kinases, covalent docking, covalent inhibitors, Michael-acceptor, natural products, reactive cysteine, AutoDockFR, EGFR

## Abstract

Tyrosine kinases (TKs) and cyclin-dependent kinases (CDKs) contain reactive cysteines that can be exploited by targeted covalent inhibitors. In this exploratory computational study, we asked whether selected natural-product-like (NP-like) electrophiles bearing Michael-acceptor (MA) motifs could adopt geometries consistent with covalent approaches to these cysteines, in a manner analogous to approved covalent TKIs. Using AutoDockFR with cysteine-centered grids and explicit side-chain flexibility, we performed pocket-focused, within-receptor covalent docking for EGFR, VEGFR2/KDR, PDGFRβ (via PDGFRα surrogate), BTK, CDK7, and CDK12. Reference inhibitors (osimertinib–EGFR, ibrutinib–BTK, THZ1–CDK7, and THZ531–CDK12) reproduced the expected geometries and served as internal controls. NP-like electrophiles (parthenolide, withaferin A, celastrol, and curcumin as a low-reactivity geometry probe) displayed pocket-compatible orientations in several targets, particularly EGFR and BTK, suggesting feasible pre-reaction alignment toward the reactive cysteine. Although no quantitative affinity was inferred, the consistent geometric feasibility supports their potential as structural templates for covalent-binding natural scaffolds. These results provide a qualitative, structure-based rationale for further chemoproteomic and enzymatic validation of NP-derived or hybrid compounds as potential leads in cancer therapy, expanding covalent chemical space beyond existing synthetic scaffolds.

## 1. Introduction

Tyrosine kinases (TKs) are validated oncogenic drivers and drug targets in solid and hematologic malignancies. Dysregulated TK signaling rewires proliferation, survival, and tumor microenvironment interactions, and can secondarily reshape cellular metabolism and nutrient use [1,2,3,4,5].

Parallel to TKs, transcription-related cyclin-dependent kinases (CDKs) integrate cell-cycle and transcriptional control and are increasingly druggable in cancer [4,5,6,7].

Across these enzyme families, pocket-facing cysteines near the ATP site have emerged as actionable residues for targeted covalent inhibitors (TCIs), enabling durable target occupancy and, in some settings, improved resistance profiles [2,8,9,10].

A prominent medicinal–chemistry strategy exploits Michael-acceptor (MA) electrophiles—most commonly acrylamides—positioned so that after noncovalent recognition, the electrophile is pre-organized to approach a reactive cysteine. Clinically validated precedents include EGFR C797 and BTK C481, where covalent TKIs achieve robust target engagement through this pre-reaction geometry [8,9,10]. At the same time, the class faces well-known challenges: resistance mutations at or near the reactive cysteine, pocket-dependent feasibility, and the need to tune intrinsic electrophile reactivity to balance on-target covalency against off-target liabilities [11].

Beyond fully synthetic scaffolds, many natural products (NPs) natively present MA-like electrophiles—e.g., α,β-unsaturated carbonyls (enones) or quinone-methide motifs—that can react with protein thiols and modulate signaling nodes [12,13].

Multiple reports show NP electrophiles covalently engaging cysteine-bearing proteins and altering kinase-centric pathways in cancer models [14,15].

Concurrently, chemoproteomic maps of the human cysteinome (including kinases) and advances in covalent docking provide structural context to ask which cysteines are ligandable and how an electrophile might be pre-organized within a given ATP pocket [16,17,18].

Together, these observations motivate a focused question: can selected NP-like electrophiles realize the same pocket-organized approach to reactive TK/CDK cysteines that underpins approved covalent TKIs—thereby offering alternative core chemotypes or complementary selectivity profiles? Because NP electrophiles can also be promiscuous if poorly pre-organized, pocket-level assessment (not pathway-level claims) is essential.

Here, we therefore set out to generate within-receptor, pocket-centric structural hypotheses about where and how Michael-acceptor (MA) chemotypes can realize geometrically plausible pre-reaction approaches to reactive cysteines in oncology-relevant kinases—EGFR, VEGFR2/KDR, PDGFRβ, BTK, CDK7, and CDK12—using AutoDockFR 1.0 with cysteine-centered grids and explicit side-chain flexibility. To anchor interpretation, each native target is paired with its reference covalent inhibitor (osimertinib, ibrutinib, THZ1, and THZ531), while a small set of natural-product-like bearing Michael-acceptor motifs such as parthenolide, withaferin A, and celastrol, with curcumin included only as a low-reactivity geometric probe, is evaluated exploratorily.

By pinpointing where MA chemotypes can plausibly approach reactive cysteines in key cancer kinases, this study highlights pockets most likely to support covalent engagement by NP-derived or hybrid scaffolds. If experimentally confirmed, these hypotheses could expand covalent chemical space, offer alternative selectivity to tackle resistance, and guide recognition-first designs that complement current TKIs in cancer therapy.

## 2. Results and Discussion

### 2.1. Reactive Cysteine Pocket Overview

**Figure 1:**
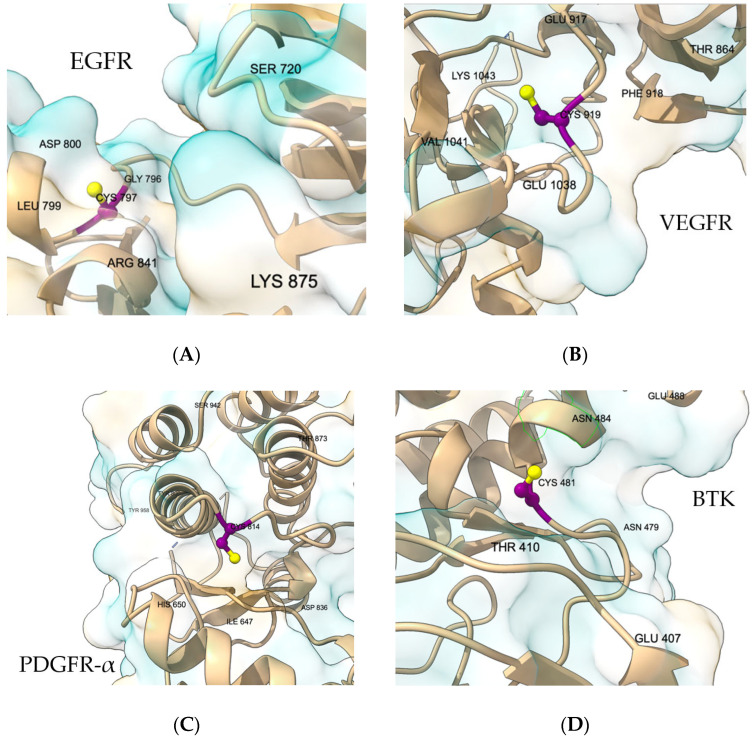

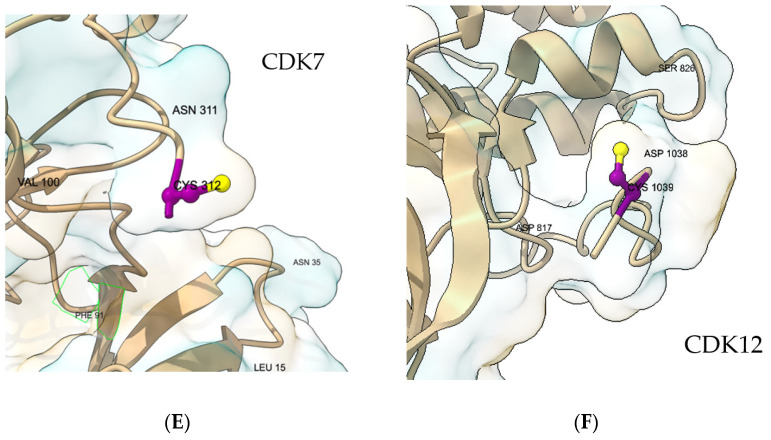
Reactive cysteine pockets across the kinase panel (**A**–**F**). Overall views and pocket insets; cysteine side chain in magenta (Sγ yellow). The docking box spans ATP site + covalent-reach region; proximal residues labeled. Images prepared in UCSF ChimeraX 1.9.

We first mapped the cysteine for each kinase within its ATP-site context to confirm pocket accessibility, hinge proximity, and front-cleft continuity. Figure 1 shows that, across targets, the reactive sulfur is pocket-facing and ATP-accessible with trajectories consistent with the Michael-acceptor approach; these views guided grid placement and the selection of locally flexible side chains used for docking. In EGFR, C797 lies in the front cleft adjacent to the hinge and gatekeeper region (Figure 1A), a geometry that supports MA approach while preserving canonical hinge recognition. In VEGFR2/KDR, C919 sits at the lip of the ATP site near the hinge-proximal front pocket, with features consistent with a type-I recognition pattern [19]. (Figure 1B). For PDGFRβ, a complete pocket-appropriate model was unavailable; the pocket view therefore derives from PDGFRα (PDB 5GRN) [20], where Cys822 corresponds to PDGFRβ Cys814 and the docking box covers the ATP pocket and adjacent cleft (Figure 1C). In BTK, C481 [21] is positioned in the front pocket with local features that match known covalent-engagement geometries (Figure 1D). In CDK7, C312 is ATP-site accessible with a clear hinge context and local features used to guide pose selection [22] (Figure 1E). In CDK12, C1039 is pocket-facing in the kinase domain [23] with a geometry consistent with prior covalent designs (Figure 1F). These pocket views provide the structural context for the docking analyses that follow, in which outputs are interpreted within each receptor and one representative pose per receptor–ligand pair is reported. Quantitative pocket-volume calculations were not performed, as the covalent-docking analysis was based on the local geometry around the reactive cysteine (Sγ accessibility and feasible approach orientation), which is not well captured by global volumetric descriptors. Therefore, pocket volume was not used as a selection or comparison criterion.

Cysteine accessibility was evaluated qualitatively by inspecting the local pocket environment around the reactive Cys residues in each kinase, considering side-chain exposure, steric occlusion by adjacent residues (e.g., gatekeeper or P-loop positions), and proximity to the ATP-binding cleft. This type of pocket-level structural inspection is standard in covalent inhibitor assessments and allows for consistent comparisons across class I and class III kinases despite structural diversity. No solvent-accessible surface calculations were performed, as the objective was to classify whether each cysteine was sterically reachable within the modeled binding pocket.

### 2.2. Synthetic Reference Ligands

**Figure 2:**
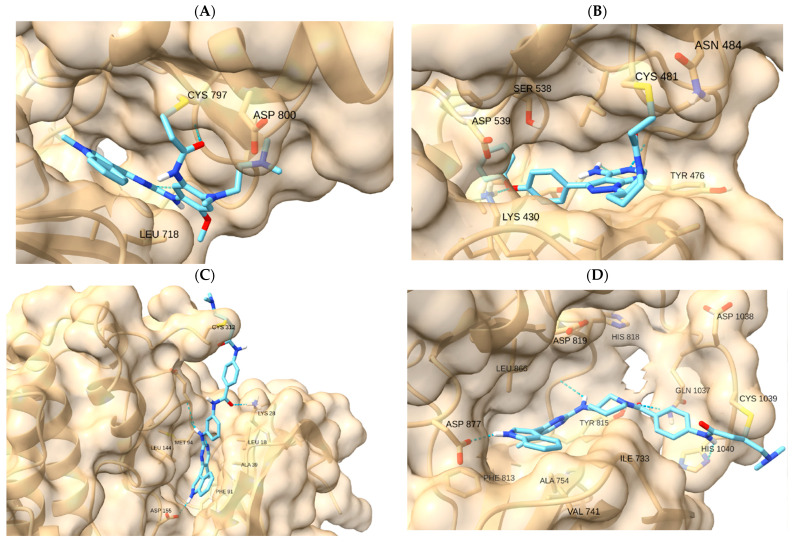
Reference covalent controls illustrate pre-reaction geometry within each native target. Poses show hinge recognition and orientation of the electrophilic center toward the reactive cysteine (Sγ): (**A**) EGFR–osimertinib (C797), (**B**) BTK–ibrutinib (C481), (**C**) CDK7–THZ1 (C312), and (**D**) CDK12–THZ531 (C1039). Boxes were centered on Sγ and included locally flexible side chains. Images prepared in UCSF ChimeraX 1.9.

To verify pocket geometry and cysteine reach, we used reference covalent inhibitors (osimertinib–EGFR, ibrutinib–BTK, THZ1/YKL-5-124–CDK7, and THZ531–CDK12). These controls are qualitative and ensure that each pocket model supports feasible pre-reaction orientation and hinge recognition (Table 1; Figure 2). Numerical outputs were used only to order poses within the same receptor prior to visual inspection; no absolute-affinity inferences or cross-target score comparisons were made.

Figure 2 displays representative reference poses. For EGFR, osimertinib—with afatinib/neratinib noted as historical comparators—benchmarks the hinge-proximal approach to C797. For BTK, ibrutinib anchors the approach to C481 in the front pocket. For transcription-related CDKs, THZ1/YKL-5-124 (CDK7 → C312) and THZ531 (CDK12 → C1039) provide covalent precedents. These references are used solely as qualitative controls to verify that each pocket model supports feasible pre-reaction orientation and coherent recognition features (e.g., hinge contacts) within the same receptor. Numerical engine outputs were used only for within-receptor pose ordering; we do not infer absolute affinities or make cross-target score comparisons (Supplementary Table S1).

For VEGFR2/KDR and PDGFRβ (assessed via PDGFRα, for structures and reactive-site mapping), widely adopted covalent benchmarks are less established; here, the references primarily informed box placement and local flexibility, while exploratory evaluation focused on NP-like electrophiles.

### 2.3. NP-like Electrophiles

We then evaluated NP-like electrophiles bearing MA motifs (parthenolide, withaferin A, and celastrol; curcumin as a low-reactivity geometry probe). Motifs and rationale are summarized in Table 2 with full pose panels provided in the Supplementary Figure S1.

These chemotypes provide diverse recognition scaffolds while positioning an electrophilic center compatible with an ATP-site approach to reactive cysteines. As a low-reactivity reference, curcumin (two enone motifs; tautomeric/flexible) has been reported to form reversible adducts with cysteine-rich proteins but displays limited electrophilic reactivity; here it was used only as a geometric reference to contrast higher-reactivity scaffolds. Compounds such as piperine (conjugated amide) and astaxanthin (polyene xanthophyll) were not retained due to the lack of clear MA-driven cysteine alkylation in kinase-relevant contexts. Piperine and astaxanthin were included only as negative NP-like controls; they consistently classified as illustrative or, at best, borderline, in agreement with their lack of an appropriately oriented Michael-acceptor motif. NP electrophiles are used here solely to generate pocket-level structural hypotheses alongside synthetic benchmarks; no absolute-affinity or cross-target comparisons are drawn.

### 2.4. Local Docking on Reactive Cysteines in Cancer-Associated Kinases

Cysteine residues present distinctive opportunities and challenges in drug design due to their nucleophilic thiol group, redox sensitivity, and capacity for covalent interactions. MAs contain α,β-unsaturated groups that are highly reactive with biological nucleophiles, especially thiol groups of cysteines. Alkylation is a post-translational modification (PTM) that adds an alkyl group to the thiol group of a cysteine residue, altering the structure and function of proteins, affecting the function of oncogenes and/or transcription factors (TF), and ultimately triggering or inhibiting pathological responses [24].

MA receptor activation is indeed a promising strategy in cancer therapy, offering potential benefits for improved efficacy and reduced toxicity compared to conventional treatments. The chemical structure of MAs can be modified to enhance their selectivity and reactivity, allowing for the development of more effective and precise cancer drugs. Research has shown that MAs can slow tumor growth and improve overall outcomes in cancer patients [6]. We therefore examined, within each receptor, whether representative ligands could adopt poses that satisfy two qualitative cues: (i) orientation of the electrophilic center toward Sγ and (ii) preservation of recognition features (e.g., hinge contacts and coherent subpocket occupancy). Numerical engine outputs were used only for within-receptor pose ordering; we do not infer absolute affinities or make cross-target comparisons. Native covalent references reproduced the expected pocket geometry in their respective targets (osimertinib–EGFR C797; ibrutinib–BTK C481; THZ1–CDK7 C312; THZ531–CDK12 C1039), supporting the docking setup and pose-selection rubric (Figure 2A–D).

NP-like electrophiles showed target-dependent feasibility. Across EGFR and BTK, several NP-like chemotypes (e.g., parthenolide, withaferin A, and celastrol; curcumin as a low-reactivity probe) could be arranged to approach Sγ while retaining partial hinge recognition, typically classified as borderline, due to incomplete enclosure or suboptimal alignment. In VEGFR2/KDR and the CDKs, tighter or more polar ATP pockets reduced simultaneous satisfaction of both cues, shifting calls toward borderline or context-only. This permissive-vs-constrained pattern mirrors what is seen with designed covalent references and provides a pocket-level rationale for where NP-derived or hybrid scaffolds are most likely to succeed (Table S1).

EGFR (C797): Reference osimertinib exhibited canonical hinge recognition and feasible C797 approach. NP-like scaffolds could be oriented toward Sγ while keeping at least partial hinge contacts; curcumin served as a geometry probe with typical borderline calls.

BTK (C481): Ibrutinib confirmed front-pocket geometry. Selected NP-like scaffolds achieved Sγ approach with recognition features visible at the pocket lip; feasibility was generally borderline without full enclosure.

VEGFR2/KDR (C919): The narrower, more polar pocket limited simultaneous Sγ approach and hinge recognition for NP-like scaffolds; calls were mainly borderline or context-only.

PDGFRβ (via PDGFRα 5GRN; C814): Mapping of the reactive site was conserved. NP-like poses illustrated pocket context; feasibility leaned on the borderline.

CDK7 (C312) and CDK12 (C1039): Covalent references matched precedent. NP-like scaffolds encountered geometric constraints (shallower or differently polarized clefts), yielding mostly borderline calls.

Collectively, these outcomes identify where MA-type chemotypes can plausibly approach reactive cysteines and which pockets are more likely to support covalent engagement by NP-derived or hybrid cores. They provide structural hypotheses to guide experiment-first validation (e.g., targeted chemoproteomics or covalent labeling) rather than claims about affinity, kinetics, or efficacy.

### 2.5. Exploratory NP-like Outcomes

**Figure 3:**
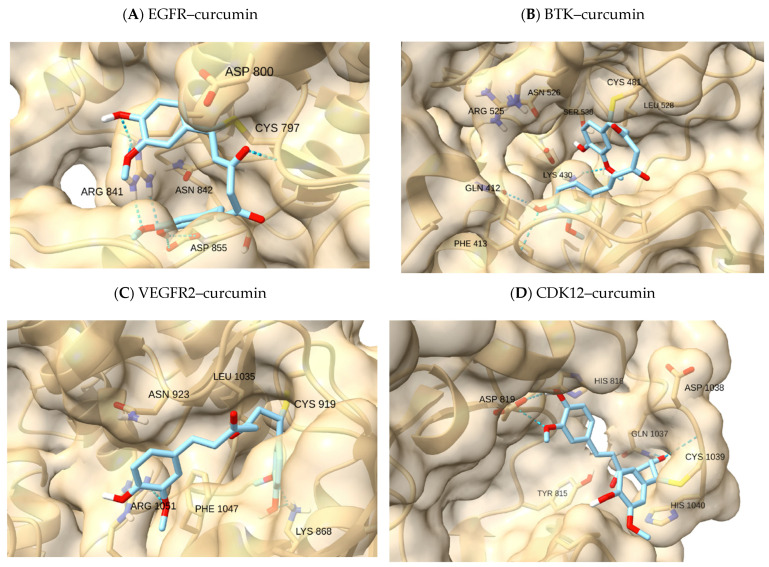
Curcumin as an NP-like geometry probe across kinase pockets. Representative docking poses of curcumin illustrate pocket-dependent feasibility according to two qualitative cues: (i) orientation of the electrophilic β-carbon (enone) toward the reactive cysteine sulfur (Sγ, yellow) and (ii) preservation of hinge recognition. (**A**) EGFR (C797)—geometry-consistent: both cues are satisfied, with hinge contacts and Sγ approach in the front cleft. (**B**) BTK (C481)—geometry-consistent to partially consistent: Sγ approach with partial hinge engagement. (**C**) VEGFR2/KDR (C919)—partially consistent: the narrower, more polar pocket limits simultaneous satisfaction of both cues. (**D**) CDK12 (C1039)—partially consistent: shallow/polar features constrain alignment (analogous behavior observed for CDK7 C312). Grids were centered on Sγ with local side-chain flexibility. Images were prepared in UCSF ChimeraX 1.9.

Building on the pocket overview and reference controls, we assessed NP-like electrophiles for geometries consistent with pre-reaction approaches to the reactive cysteine while preserving recognition features. Table 3 summarizes the within-receptor qualitative calls (feasible/borderline/illustrative). Qualitative “covalent-approach calls” reflect the geometric plausibility of pre-reaction alignment between the electrophilic center and the cysteine Sγ atom, as assessed from top-ranked docking clusters. “Feasible” indicates spatial orientation consistent with known covalent complexes; “borderline” denotes partial or suboptimal alignment; and “illustrative” refers to non-covalent or geometrically distant configurations. Across the set, EGFR (C797) and BTK (C481) were the most permissive pockets for NP-like chemotypes: sesquiterpene lactones and steroidal enones could be arranged with the electrophilic center oriented toward Sγ while retaining at least partial hinge recognition, yielding feasible or borderline calls depending on depth of enclosure. In VEGFR2/KDR (C919) and PDGFRβ (modeled via PDGFRα 5GRN), narrower/more polar clefts reduced simultaneous satisfaction of both cues, shifting outcomes toward the borderline. For CDK7 (C312) and CDK12 (C1039), covalent references reproduced the precedent, whereas NP-like scaffolds faced geometric constraints and were typically borderline. Table 3 summarizes qualitative calls; Figure 3 provides a single NP-probe example (curcumin) to illustrate pocket-dependent feasibility. Within each receptor, docking energies followed the expected qualitative ranking (reference covalent inhibitors < NP-like electrophiles < low-reactivity control). Numerical values for all complexes are reported in Supplementary Table S1.

Taken together, these findings point to EGFR and BTK as the most promising contexts for NP-derived or hybrid covalent designs and suggest that pocket polarity/shape—rather than electrophile type alone—govern feasibility. These qualitative hypotheses are intended to guide experiment-first follow-up (e.g., targeted chemoproteomics or covalent labeling) rather than imply potency or efficacy.

Within-receptor controls reproduced the precedent—osimertinib in EGFR (C797), ibrutinib in BTK (C481), THZ1 in CDK7 (C312), and THZ531 in CDK12 (C1039) all scored as *feasible*. NP-like probes showed pocket-dependent behavior: curcumin reached *borderline* geometry in EGFR and BTK, and astaxanthin was *borderline* in EGFR; piperine reached *borderline* in VEGFR2/KDR’s narrower cleft. In PDGFRβ (via PDGFRα), only ibrutinib achieved a *borderline* approach (Table 3). Sunitinib, a reversible non-covalent TKI, systematically classified as ‘illustrative’ in all targets, was used as an internal negative control to represent non-covalent, context-only binding. These ‘illustrative’ classifications explicitly serve as negative cases, where the ligand fails to achieve a productive Sγ → electrophile alignment despite correct pocket placement.

### 2.6. Pocket-Level Interpretation Across Receptors

The kinase pockets examined in this study differ in well-established architectural features that influence ligand orientation, cysteine accessibility, and the likelihood of accommodating a productive covalent approach. Such differences are extensively described in structural studies of kinase families, which highlight how gatekeeper size, front-pocket depth, the continuity of hydrophobic channels, and P-loop mobility collectively determine whether a pocket behaves as “narrower,” “more polar,” or “more permissive” in qualitative terms [25,26,27]. In our analysis, these canonical structural determinants —rather than quantitative pocket-volume metrics—guided the pocket-level interpretation of docking outcomes. This allowed us to contextualize the observed feasible, borderline, or illustrative orientations within the characteristic topology of each receptor.

#### 2.6.1. EGFR

Within EGFR, the benchmark pose for osimertinib reproduced the expected hinge anchoring with acrylamide oriented toward C797, consistent with the reference geometry and providing an internal control for the protocol. Among NP-like probes, astaxanthin populated the hydrophobic cleft and presented terminal polar contacts at either end of the channel—an arrangement consistent with reports that deep hydrophobic enclosure plus terminal polar interactions stabilize ligands in EGFR’s ATP site [28,29]. Although astaxanthin is not a canonical MA electrophile, its elongated scaffold illustrates how hydrophobic pre-organization can enable a borderline approach geometry. THZ1, despite a distinct scaffold, adopted a broadly similar placement in the apolar channel (shape complementarity/lipophilic fit), while ibrutinib and sunitinib showed hinge-region hydrogen bonds typical of ATP-competitive recognition in EGFR [30]. As expected, osimertinib provided the feasible covalent-control geometry; the remaining non-native covalent references served as illustrative comparators for pocket context (Table 3).

#### 2.6.2. VEGFR2

In VEGFR2, the binding cleft is narrower and more polar, and several ligands that positioned well in EGFR remained partially solvent-exposed here. This limited simultaneous satisfaction of the two cues (Sγ approach + hinge recognition), often yielding borderline or illustrative outcomes—mirroring prior observations that hydrophobic enclosure is particularly important for stabilization in VEGFR2 [31]. Piperine, being compact and semi-rigid, sometimes achieved tighter packing with Lys868/Arg1051 engagement, consistent with the notion that smaller ligands can perform better in constrained/polar environments [32]. By contrast, curcumin and osimertinib tended to show suboptimal placement relative to key catalytic features in our qualitative review (Table 3).

#### 2.6.3. PDGFR

For PDGFR, NP-like probes generally produced illustrative or borderline arrangements, aligning with the literature that the PDGFRα pocket often prefers more rigid, heteroaromatic scaffolds that can fill the hydrophobic core and satisfy specific polar contacts [33]. Ibrutinib—a rigid, polycyclic scaffold—achieved the clearest pocket complementarity among the non-native comparators, consistent with the deep-cleft occupancy seen for rigid heterocycles in PDGFR contexts. Occasional hinge/backbone contacts observed with osimertinib suggest potential cross-pocket compatibility, in line with documented cross-reactivity of some TKIs across RTKs [34] (Table 3).

#### 2.6.4. BTK

For BTK, the covalent reference ibrutinib reproduced the canonical C481 approach with hinge anchoring (feasible), consistent with the structural precedent [35]. Hydrophobic scaffolds—astaxanthin in particular—could occupy the elongated front pocket and present a plausible orientation toward C481 while relying predominantly on apolar contacts (borderline), a behavior coherent with the pocket’s tolerance for shape-complementary hydrophobes [36]. Curcumin and piperine tended to engage more superficially at the pocket entrance, giving borderline or illustrative calls depending on the visible recognition features (Table 3).

#### 2.6.5. CDK7 and CDK12

In CDK7, the validated covalent reference THZ1 reproduced precedent geometry at C312 (feasible), whereas larger or more flexible NP-like scaffolds often faced topological/polar constraints consistent with a shallower or more selective ATP cleft [37,38,39]. In CDK12, THZ531 recapitulated the expected pre-reaction arrangement at C1039 (feasible), and we occasionally observed geometry-compatible placements for osimertinib (non-native) that echo prior reports of ATP-competitive scaffolds cross-reacting with CDKs under certain conditions [23,40]. NP-like probes were generally borderline here, aligning with evidence that CDK12 selectivity benefits from scaffold rigidity and precise hinge alignment [41,42] (Table 3).

### 2.7. Synthesis Across Targets

Taken together, EGFR (C797) and BTK (C481) were the most permissive pockets for NP-like chemotypes, frequently achieving feasible or borderline pre-reaction orientations (depending on depth of enclosure and hinge coherence), while VEGFR2/KDR (C919) and PDGFRβ (via PDGFRα mapping) tended to restrict the simultaneous satisfaction of both cues. In CDK7 and CDK12, native covalent references anchored feasibility as expected, whereas NP-like scaffolds were more often borderline. These qualitative observations reinforce a pocket-first view: pocket shape and polarity—more than electrophile identity per se—govern whether a scaffold can realize a geometry consistent with covalent engagement. The resulting structural hypotheses are intended to guide experiment-first validation (e.g., cysteine-targeted chemoproteomics or covalent labeling) rather than imply rank-ordering of affinity, kinetics, or efficacy.

To ensure transparency and reproducibility, all docking poses and numerical outputs are provided in the Supplementary Information.

Figures S1–S6 display all poses obtained from the top-ranked clusters (RMSD ≤ 2.0 Å) for each receptor–ligand pair, with the reactive cysteine residues highlighted in yellow.

Supplementary Table S1 lists the corresponding docking scores (ADFR energy units, kcal/mol). These values are provided solely for within-receptor pose ranking and are not intended for quantitative affinity comparison across ligands or targets.

## 3. Materials and Methods

### 3.1. Selection of Kinase Targets

We prioritized EGFR, VEGFR2/KDR, PDGFRβ, BTK, CDK7, and CDK12 based on (i) their well-established roles in oncogenic signaling and therapy, and (ii) the presence of the literature-reported reactive cysteines that are spatially accessible from the ATP site and thus are compatible with covalent modulation. Together, these enzymes span receptor and non-receptor tyrosine kinases as well as transcription-related CDKs, covering pathways central to proliferation, angiogenesis, immune activation, transcriptional control, and DNA-damage responses.

Candidate cysteines were identified from experimental reports and curated annotations describing reactive/ligandable residues, then were inspected in the context of hallmark kinase elements (hinge, gatekeeper vicinity, P-loop, DFG and A-loop, and αC-helix). Residues were retained when their thiol groups are solvent- and pocket-accessible and geometrically positioned to be approached from the ATP site. Selection also required high-quality protein structures encompassing the relevant pocket (see Table S1 for PDB IDs, chains, and residue indices).

For PDGFRβ, where a complete pocket-appropriate structure was unavailable, we used PDGFRα (PDB: 5GRN) as a structural surrogate. Sequence alignment and a local pocket comparison indicate conservation at the reactive cysteine site (PDGFRα Cys822/PDGFRβ Cys814), supporting the transfer of pocket geometry for qualitative assessment. The structural similarity between PDGFRα and PDGFRβ within the intracellular kinase domain is well supported by crystallographic and modeling studies. In particular, superposition of the PDGFRβ kinase domain onto the PDGFRα template (PDB ID: 5GRN) shows an RMSD of 0.436 Å, confirming their nearly identical topology and ATP-site architecture [43]. This strong conservation supports the use of PDGFRα as a reliable structural surrogate for qualitative geometry-based assessments. This panel enables a comparative, pocket-level evaluation of covalent approach across kinase classes while anchoring interpretation to oncology-relevant targets.

### 3.2. Selection of Synthetic Alkylating Agents

We curated a small set of Michael-acceptor (MA)-bearing covalent kinase ligands to serve as reference controls for pocket geometry and cysteine reach. A selection prioritized compounds with (i) a documented covalent mechanism to a reactive cysteine in the ATP-site environment, (ii) structural support (co-crystal structures and/or cysteine-to-alanine/serine mutational evidence), (iii) oncological relevance, and (iv) diverse hinge-binding scaffolds to sample recognition patterns across kinase folds.

Accordingly, we included osimertinib and afatinib/neratinib (EGFR/ErbB family; acrylamide MA positioned toward the hinge-proximal cysteine), ibrutinib (BTK; acrylamide to C481), and covalent CDK chemotypes THZ1/YKL-5-124 (CDK7) and THZ531 (CDK12/13). These ligands are used solely as qualitative benchmarks to contextualize pocket-feasible covalent orientations in each receptor; they are not intended for absolute affinity estimation or cross-target score comparisons.

### 3.3. Selection of Natural Electrophilic Compounds

We assembled a focused set of natural-product (NP) electrophiles bearing Michael-acceptor (MA) motifs with the support of the literature for cysteine alkylation and oncology-relevant signaling. Selection emphasized (i) established electrophilic functionality (e.g., α,β-unsaturated carbonyls/enones and quinone-methide systems), (ii) reports linking the scaffold to kinase-centric biology or covalent protein modification, and (iii) chemical compatibility with pocket-level covalent engagement (MA placement amenable to approach from the ATP site). The intent is to sample diverse NP chemotypes that plausibly pre-organize MA motifs in kinase pockets, without implying clinical positioning or absolute affinities.

Accordingly, the NP set included the following:

- Parthenolide (sesquiterpene lactone; α-methylene-γ-lactone/enone): widely used as a model MA electrophile; reported to modulate EGFR-related signaling and to alkylate protein cysteines in cancer contexts.
- Withaferin A (steroidal lactone; enone): documented covalent targeting of BTK-family cysteines and other thiol-dependent proteins in hematologic models.
- Celastrol (triterpenoid quinone-methide): electrophile with reports of EGFR-axis modulation/sensitization and cysteine-directed reactivity.

Curcumin was included solely as a low-reactivity control to assess geometric feasibility. In addition to parthenolide, withaferin A, celastrol, and curcumin (used as a low-reactivity geometric control), we also retained piperine and astaxanthin as NP-like scaffolds used solely as negative geometric controls. They lack a suitably oriented electrophilic warhead and systematically failed to achieve a productive Sγ approach and are therefore included only as illustrative ‘context-only’ examples.

### 3.4. Protein Preparation

Protein coordinates were taken from the PDB entries. For PDGFRβ, PDGFRα (5GRN) served as a structural surrogate based on conservation of the reactive-site mapping (PDGFRα Cys822/PDGFRβ Cys814). Structures were processed in UCSF ChimeraX 1.9 [44]: non-protein entities were removed (retaining only clearly resolved waters mediating hinge/catalytic H-bonds), alternate locations were set to the highest-occupancy conformer, hydrogens were added, and default pH 7.4 protonation/tautomer rules were applied. Protonation states of key catalytic residues (β3 Lys, αC-Glu, and DFG Asp) in each kinase were manually inspected in ChimeraX to ensure chemically reasonable active-site charge states at pH 7.4. The literature-reported reactive cysteine for each target was visually verified to be pocket-facing and ATP-accessible. No backbone minimization was applied.

### 3.5. Ligand Preparation

Synthetic covalent references and NP-like electrophiles were standardized (desalting/neutralization; functional-group normalization), with the literature’s stereochemistry preserved; ambiguous centers were enumerated in RDKit and the literature-consistent configuration was retained. Major microstates at pH 7.4 were selected. For curcumin, the predominant diketo tautomer at physiological pH was used, as this is considered the dominant microstate under docking conditions. Conformers were generated with RDKit ETKDG and minimized (MMFF94; UFF fallback). For MA-bearing chemotypes (e.g., acrylamides, enones, and quinone-methides), the electrophilic center intended for cysteine approach (β-carbon for enones/acrylamides; electrophilic center for quinone-methides) was annotated. Ligands were converted to PDBQT via ADFR’s prepare_ligand [45]; No artificial derivatization or warhead substitution was performed.

### 3.6. Cysteine Mapping and Structural Localization

For each receptor, the reactive cysteine was mapped within the ATP site, and pocket surfaces/meshes were inspected to confirm cavity continuity from the ATP site to cysteine Sγ. This informed grid centering and the selection of locally flexible side chains.

### 3.7. Covalent Docking

Covalent docking used AutoDockFR (ADFR) [46], derived from AutoDock [47], combining a genetic algorithm global search with local refinement on gradient-based affinity maps and receptor side-chain sampling from a rotamer library [48]. Engine outputs were used as a heuristic to order poses within the same receptor prior to visual assessment; interpretation emphasized pre-reaction geometry and recognition features, and we avoided cross-target score comparisons. AutoGridFR maps [49] were computed in a default cubic box of 22 × 22 × 22 Å centered on the reactive cysteine Sγ, spanning the ATP site and the covalent-reach region. For AutoGridFR, we used a whole-protein grid centered on the receptor and generated using the default receptor-adaptive grid dimensions, which automatically expanded to include all residues and potential reactive cysteines. The grid box encompassed the entire kinase domain, ensuring unrestricted sampling of ligand orientations. Side-chain flexibility was enabled for pocket residues (Table 4; residue numbering follows the PDB entry used). Multiple independent runs per receptor–ligand pair were clustered at 2.0 Å heavy-atom RMSD. One representative pose per pair was retained from the top-ranked cluster (within receptor), prioritizing (i) orientation of the electrophilic center toward Sγ and (ii) hinge recognition/coherent subpocket occupancy. Poses were annotated as feasible (both cues) or borderline (one cue). These annotations are descriptive and pocket-centric.

#### 3.7.1. Ligands and Reactive Centers

Ligands included reference covalent inhibitors paired to their native targets (osimertinib–EGFR, ibrutinib–BTK, THZ1–CDK7, and THZ531–CDK12) together with NP-like electrophiles bearing Michael-acceptor-type motifs (e.g., enones/quinone-methides). For MA-bearing chemotypes, the electrophilic center intended for cysteine engagement (β-carbon for acrylamides/enones; electrophilic center for quinone-methides) was annotated. Where applicable, RDKit (version 2024.03.6) was used to enumerate chemically reasonable stereoisomers/regioisomers; ligands were converted to PDBQT using ADFR prepare_ligand. No artificial derivatization was applied.

#### 3.7.2. Search Settings, Clustering, and Pose Selection

Multiple independent runs (distinct random seeds) were executed per receptor–ligand pair. For each ligand–receptor complex, approximately 20 poses were generated by the docking engine. After clustering, the lowest-energy representative pose from the dominant cluster was selected for qualitative geometric inspection. Resulting poses were clustered at 2.0 Å heavy-atom RMSD. One representative pose per receptor–ligand pair was retained from the top-ranked cluster (within receptor), prioritizing (i) orientation of the electrophilic center toward cysteine Sγ and (ii) preservation of hinge recognition and coherent subpocket occupancy. Absolute affinities were not inferred, and cross-target score comparisons were avoided.

#### 3.7.3. Qualitative Annotations

Covalent-orientation assessment was therefore performed qualitatively, based on (i) the presence of a direct line-of-sight between the ligand warhead and the reactive cysteine Sγ, (ii) the absence of steric occlusion by gatekeeper or P-loop residues, and (iii) a near-colinear approach compatible with Michael-type addition as typically described for cysteine-reactive kinase inhibitors. Within this framework, poses were annotated as *feasible* (✓) when these criteria were jointly satisfied, or *borderline* (~) when only partial alignment was observed. These qualitative annotations provide pocket-centric guidance on covalent compatibility and are intended to support subsequent experimental prioritization.

Descriptions such as ‘narrower’, ‘more polar’, or ‘more permissive’ refer to the qualitative structural features of each ATP-binding cleft, assessed from the crystallographic coordinates using ChimeraX. Pocket width was evaluated visually based on gatekeeper residue position and P-loop conformation; polarity was inferred from the distribution of charged and hydrogen-bonding residues lining the front and hinge regions; and permissiveness reflects the relative absence of steric encroachment into the front pocket or hydrophobic channel. These qualitative assessments are widely used in kinase structural analysis and do not rely on numerical pocket-volume calculations [25,26,27].

## 4. Conclusions

In this work, we conducted an exploratory, pocket-centric covalent docking analysis of synthetic and natural electrophiles targeting reactive cysteines in oncogenic kinases. By using AutoDockFR with explicit side-chain flexibility and cysteine-focused grids, we identified pose geometries consistent with pre-reaction approaches for several Michael-acceptor chemotypes. Benchmark covalent inhibitors reproduced known geometries, validating the pocket setup and confirming the method’s qualitative reliability. Among the natural products tested, parthenolide, withaferin A, and celastrol adopted feasible or borderline orientations toward the nucleophilic cysteine in EGFR, BTK and, to a lesser extent, VEGFR2 pockets, while curcumin served as a low-reactivity geometric control confirming the method’s discrimination capacity. These findings indicate that NP-derived scaffolds can achieve spatial arrangements compatible with covalent engagement, supporting their potential as experimental candidates for cysteine-targeted modulation of kinase activity. Although this study is purely computational and qualitative, it generates actionable structural hypotheses for experiment-first validation.

Future work should combine covalent chemoproteomics, enzymatic inhibition assays, and structure–activity optimization to confirm and refine these leads. If validated, such NP-like electrophiles may offer alternative or complementary selectivity profiles to current covalent TKIs and contribute to the discovery of novel anticancer therapeutics based on natural scaffolds.

## Figures and Tables

**Table 1:** Reference ligands and NP-like electrophiles considered.

Receptor	Synthetic Covalent Reference(s)	NP-like Electrophiles	Purpose
**EGFR**	Osimertinib; Afatinib/Neratinib	Parthenolide Celastrol	Qualitative pocket benchmarks + exploratory NP chemotypes
**BTK**	Ibrutinib	Withaferin A	Qualitative pocket benchmarks + exploratory NP chemotypes
**VEGFR2/KDR**	—	Parthenolide/Celastrol	Exploratory NP chemotypes
**PDGFRβ**	—	Parthenolide/Celastrol	Exploratory NP chemotypes
**CDK7**	THZ1; YKL-5-124	—	Synthetic precedent
**CDK12**	THZ531	—	Synthetic precedent

**Table 2:** NP-like electrophiles are evaluated as exploratory chemotypes. Entries summarize the Michael-acceptor motif and rationale; compounds are used to generate pocket-level structural hypotheses alongside synthetic covalent benchmarks.

NP-like Electrophile	Electrophile Motif (MA Type)	Rationale/Evidence	Role Studied
Parthenolide	α-methylene-γ-lactone (exocyclic enone)	Literature precedent for cysteine alkylation; engages signaling nodes relevant to kinases	Exploratory chemotype to probe ATP-site approach to reactive Cys
Withaferin A	Steroidal enone	Thiol reactivity reported; modulates kinase-centric pathways in cancer models	Exploratory chemotype (shape-complementary, semi-rigid core)
Celastrol	Quinone/quinone-methide–like	Electrophilic scaffold with cysteine-targeting reports; affects stress/kinase pathways	Exploratory chemotype (distinct electrophile class)
Curcumin (optional)	Two enone motifs; flexible/tautomeric	Lower intrinsic reactivity; useful to test geometry without implying potency	Low-reactivity reference for geometry only

**Table 3:** Qualitative covalent-approach calls (within receptor) reflecting pocket-level geometry (Cys Sγ approach + hinge recognition).

Receptor (Reactive Cys)/Ligand	Sunitinib ^†^	Osimertinib	Ibrutinib	THZ1	THZ531	Curcumin	Piperine	Astaxanthin
EGFR (C797)	illustrative	feasible	illustrative	illustrative	illustrative	borderline	illustrative	borderline
VEGFR2/KDR (C919)	illustrative	illustrative	illustrative	illustrative	illustrative	illustrative	borderline	illustrative
PDGFRβ (C814)	illustrative	illustrative	borderline	illustrative	illustrative	illustrative	illustrative	illustrative
BTK (C481)	illustrative	illustrative	feasible	illustrative	illustrative	borderline	illustrative	borderline
CDK7 (C312)	illustrative	illustrative	illustrative	feasible	illustrative	illustrative	illustrative	illustrative
CDK12 (C1039)	illustrative	borderline	illustrative	illustrative	feasible	illustrative	illustrative	illustrative

Piperine and astaxanthin displayed only distant, non-covalent illustrative poses. ^†^ Sunitinib is a reversible, non-covalent TKI; included here as a pocket-context comparator only.

**Table 4:** Flexible receptor side chains used in covalent docking.

Receptor	Flexible Side Chains (Residue IDs)	Numbering Basis (PDB)
BTK	Gln412, Lys430, Arg525, Asn526	5P9J
CDK12	His818, Asp819, Gln1037, Asp1038, His1046	7NXK
CDK7	Lys28, Glu95, Arg309, Asn311	6XD3
EGFR	Leu718, Asp800, Arg841, Asn842, Asp855	6JXT
PDGFRα (surrogate for β)	Lys627, His816, Arg817, Asp836	5GRN
VEGFR2	Lys838, Lys868, Lys920, Arg1032, Arg1051	3WZE

Residue numbering follows the PDB entry indicated for each receptor (EGFR 6JXT; VEGFR2 3WZE; PDGFRα 5GRN [surrogate for PDGFRβ]; BTK 5P9J; CDK7 6XD3; CDK12 7NXK).

## Data Availability

The original contributions presented in this study are included in the article/Supplementary Materials. Further inquiries can be directed to the corresponding authors.

## Supplementary Materials

The following supporting information can be downloaded at: https://www.mdpi.com/article/10.3390/ijms262311390/s1.
