# Supplementary material for: Exploratory Covalent Docking of Michael-Acceptor Natural Products at Reactive Cysteines in Cancer Tyrosine Kinases
Source: Int J Mol Sci. 2025 Nov 25;26(23):11390. doi: 10.3390/ijms262311390 (PMC12692623; doi:10.3390/ijms262311390)
Supplement: Supplementary file 1 [file ijms-26-11390-s001.zip › ijms-3830151-supplementary.pdf]

Supplementary Materials:

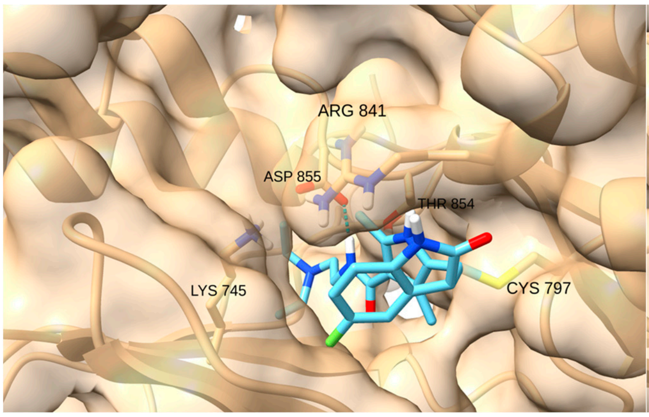

Sunitinib

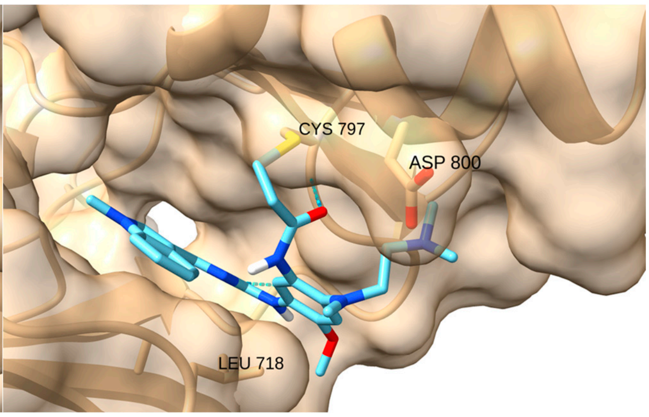

Osimertinib

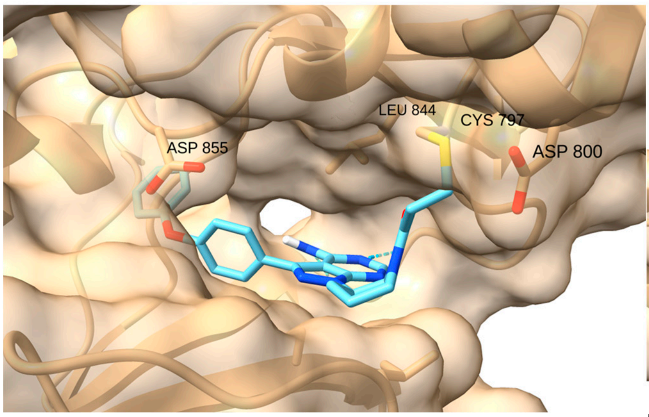

Ibrutinib

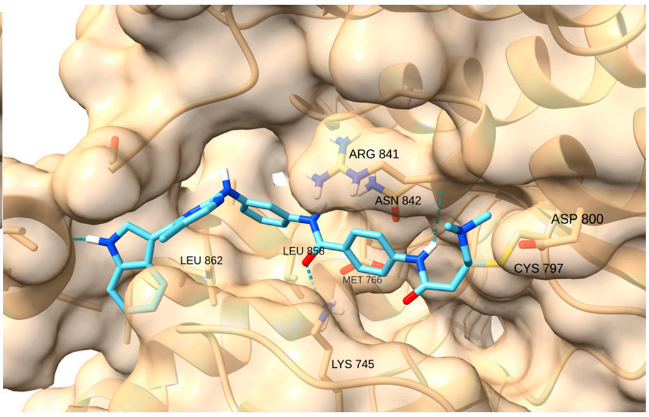

THZ1

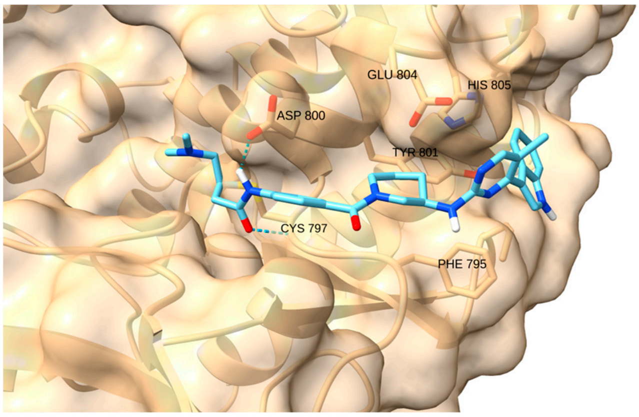

THZ531

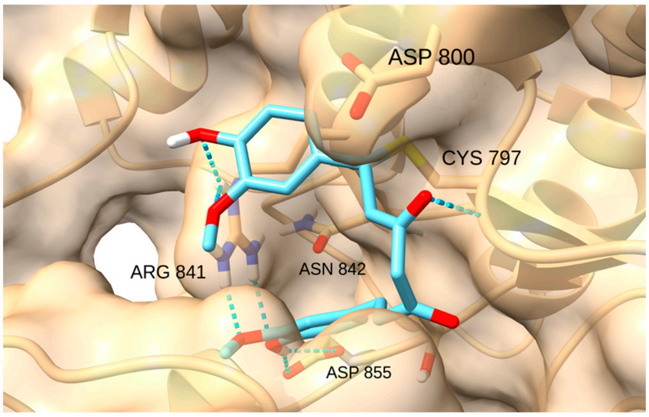

Curcumin

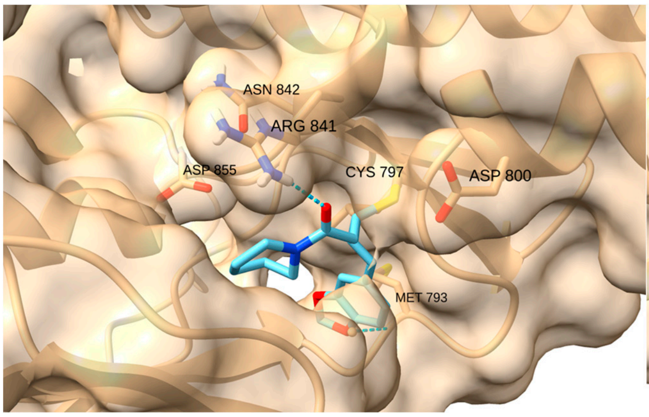

Piperine

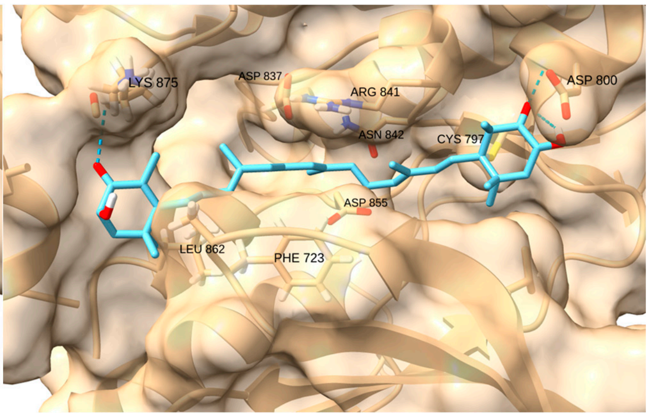

Astaxanthin

Figure S1. All docking poses for natural and reference covalent inhibitors in EGFR with ligands Sunitinib, Osimertinib, Ibrutinib, THZ1, THZ531, Curcumin, Piperine, and Astaxanthin. Reactive cysteines are shown in yellow (Sγ).

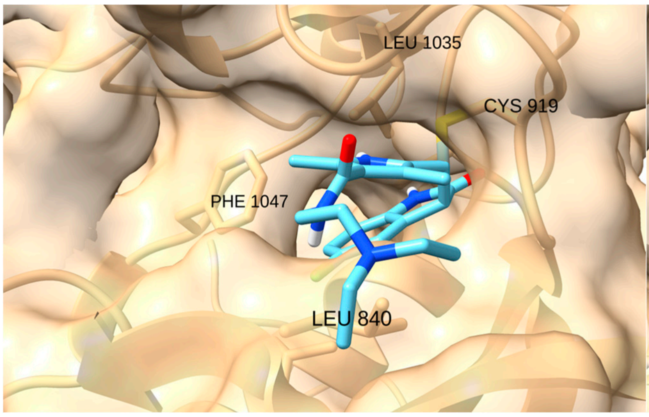

Sunitinib

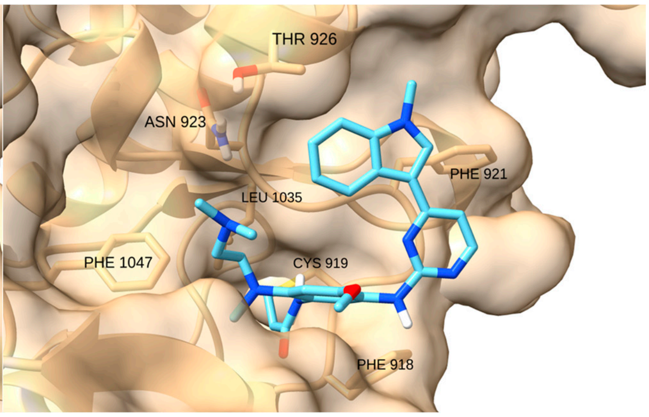

Osimertinib

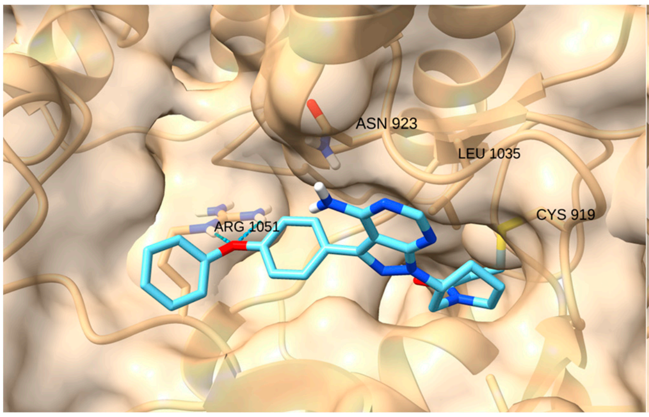

Ibrutinib

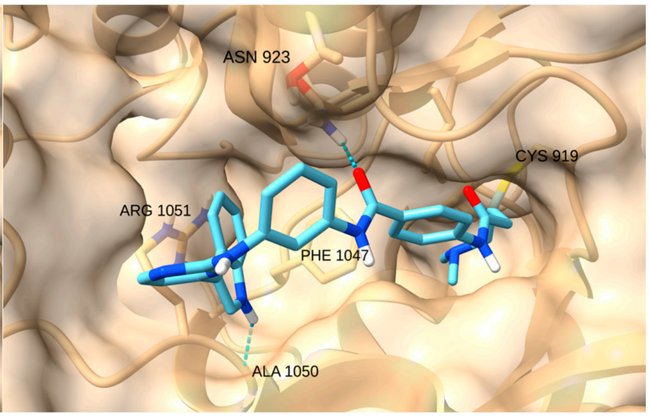

THZ1

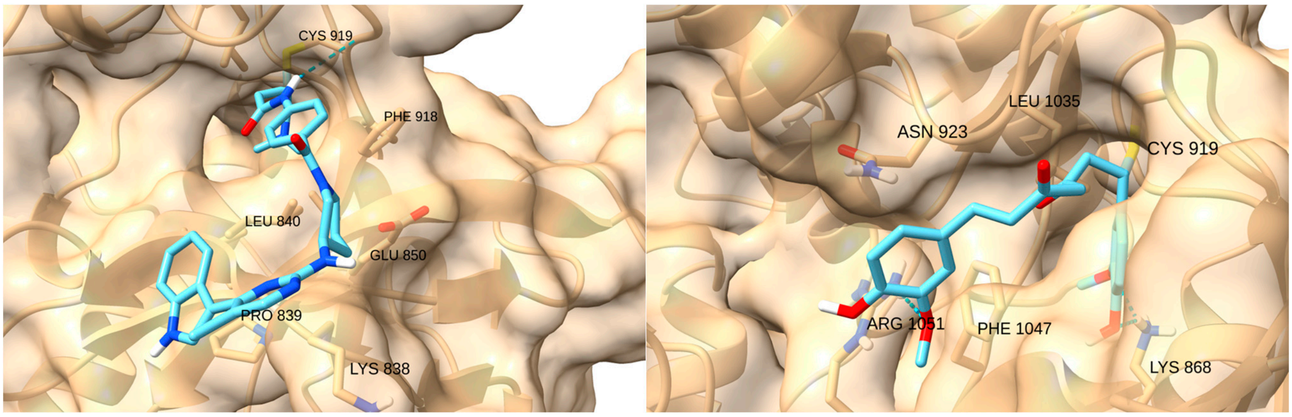

THZ531 Curcumin

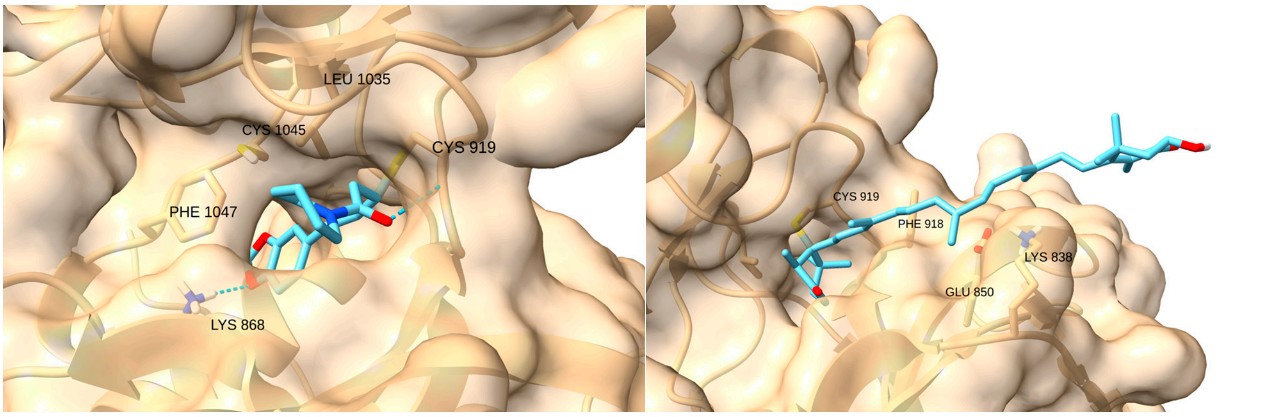

Piperine Astaxanthin

Figure S2. All docking poses for natural and reference covalent inhibitors in VEGFR2 with ligands Sunitinib, Osimertinib, Ibrutinib, THZ1, THZ531, Curcumin, Piperine, and Astaxanthin. Reactive cysteines are shown in yellow (Sγ).

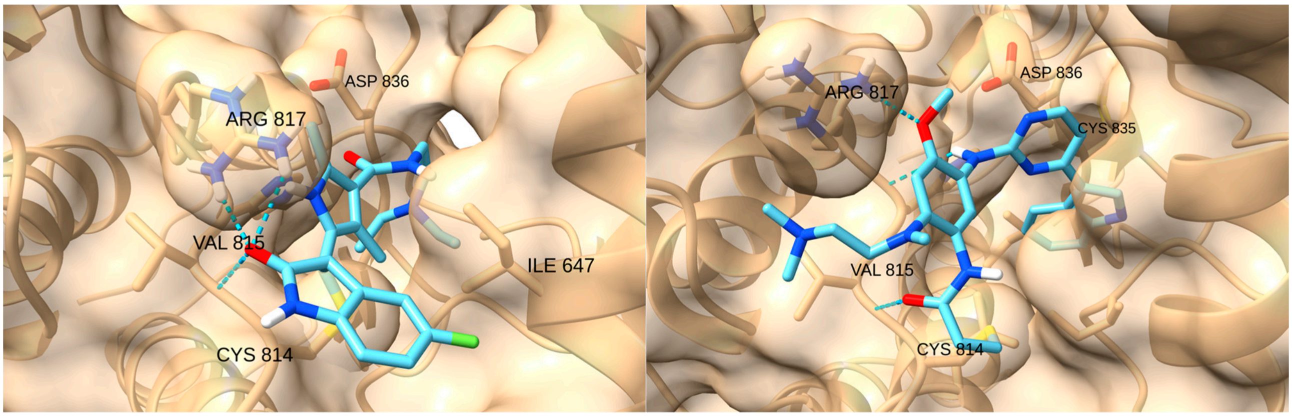

Sunitinib Osimertinib

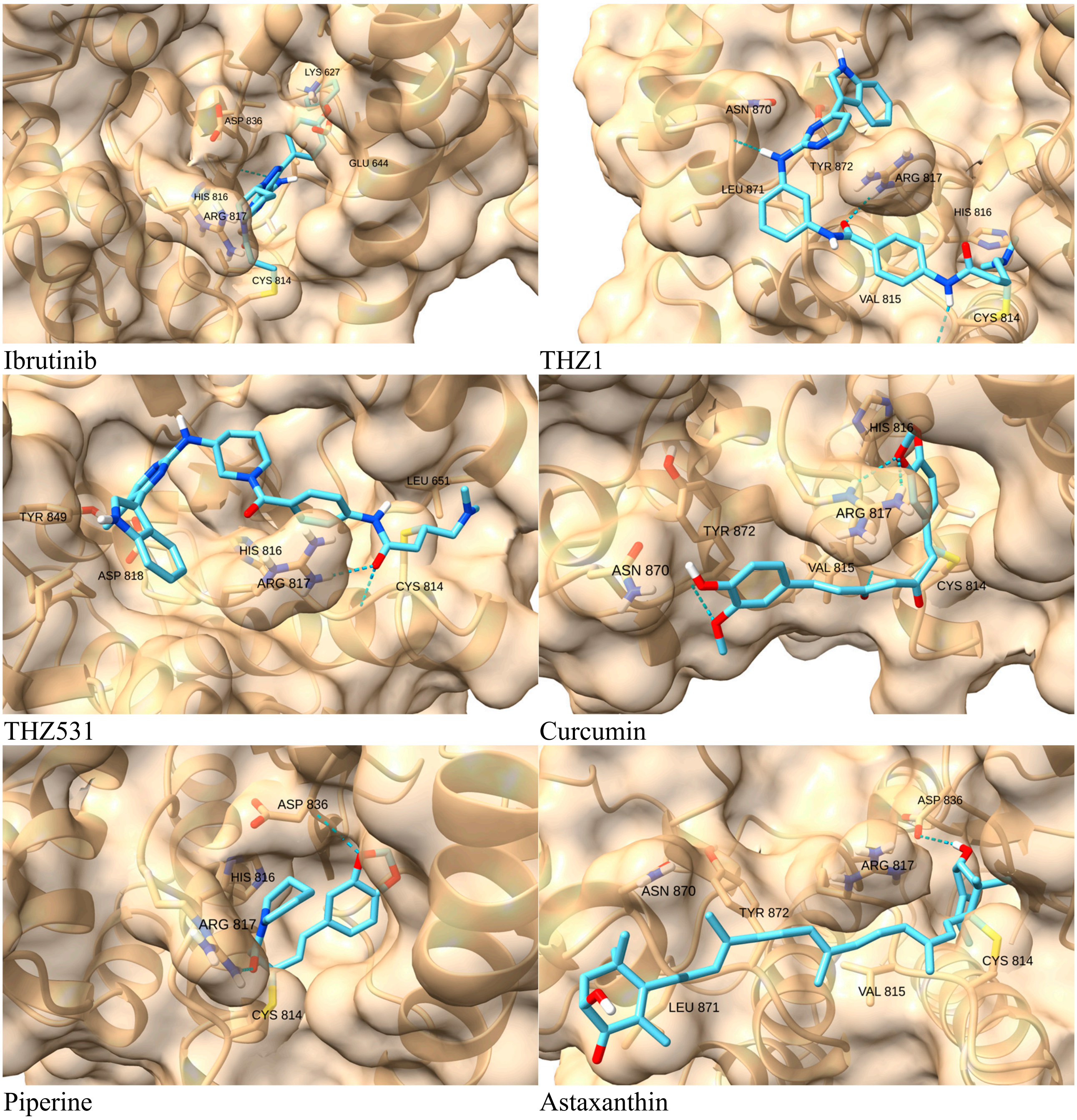

Figure S3. All docking poses for natural and reference covalent inhibitors in PDGFR with ligands Sunitinib, Osimertinib, Ibrutinib, THZ1, THZ531, Curcumin, Piperine, and Astaxanthin. Reactive cysteines are shown in yellow (Sγ).

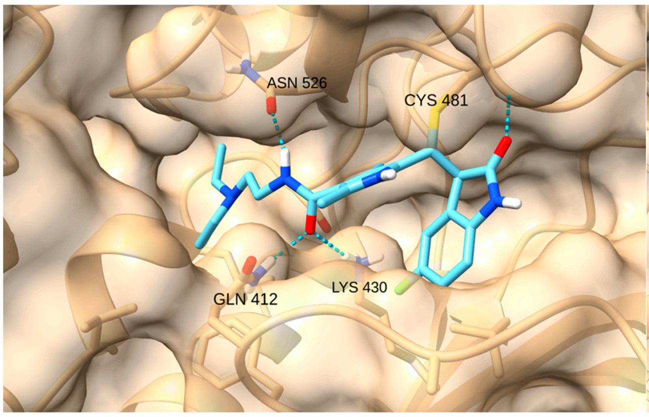

Sunitinib

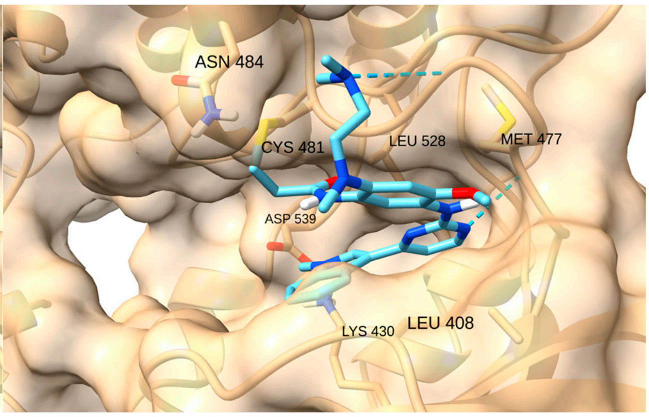

Osimertinib

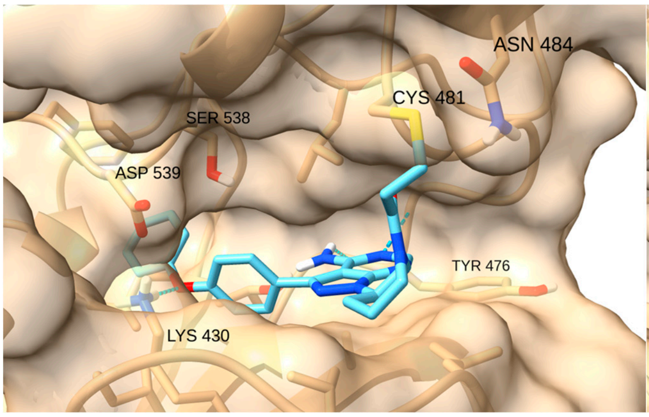

Ibrutinib

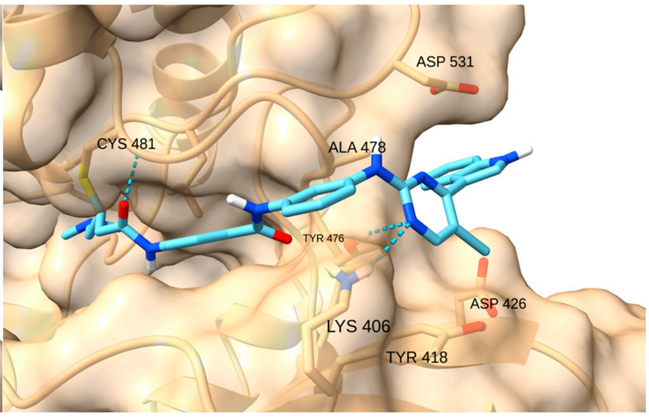

THZ1

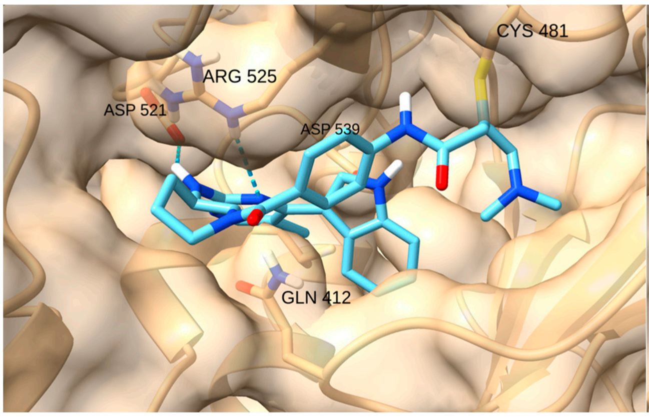

THZ531

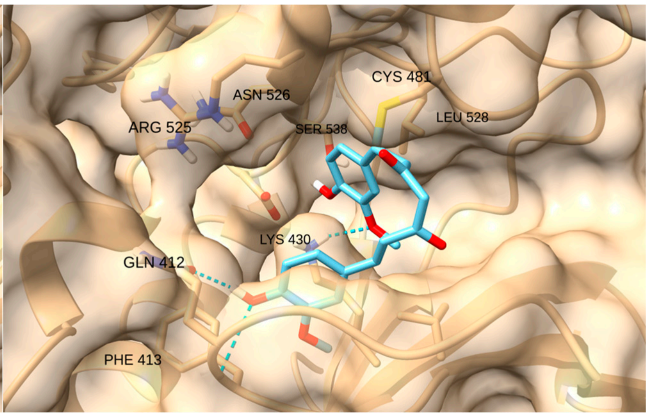

Curcumin

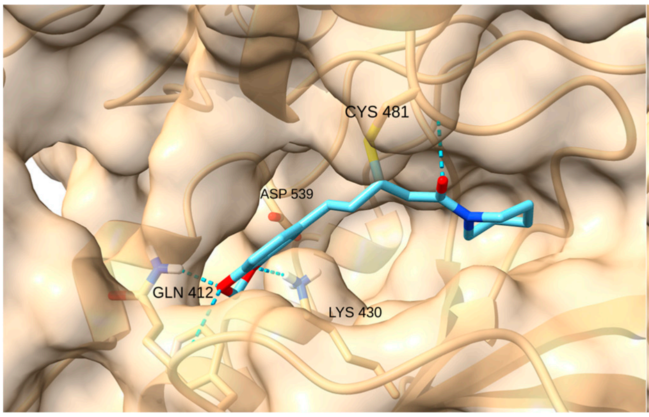

Piperine

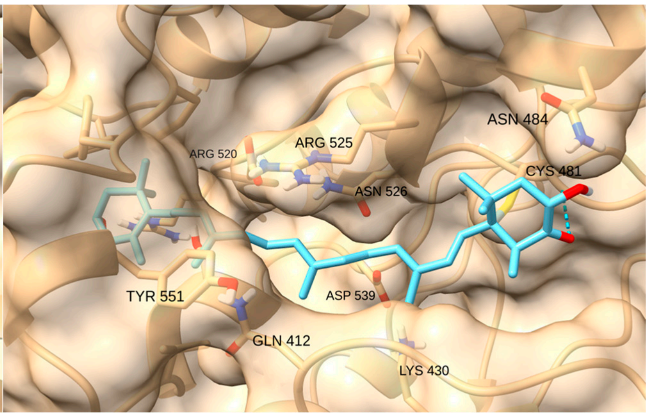

Astaxanthin

Figure S4. All docking poses for natural and reference covalent inhibitors in BTK with ligands Sunitinib, Osimertinib, Ibrutinib, THZ1, THZ531, Curcumin, Piperine, and Astaxanthin. Reactive cysteines are shown in yellow (Sγ).

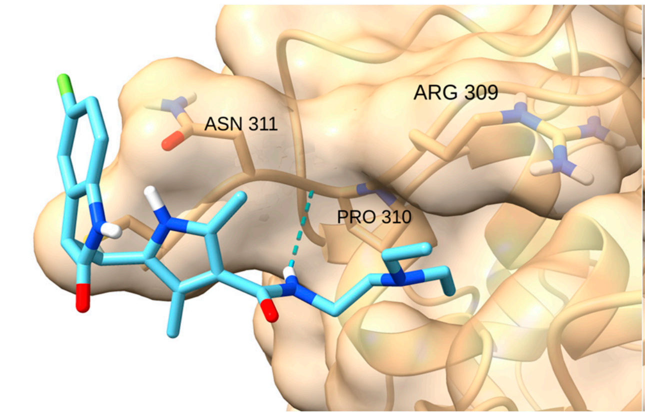

Sunitinib

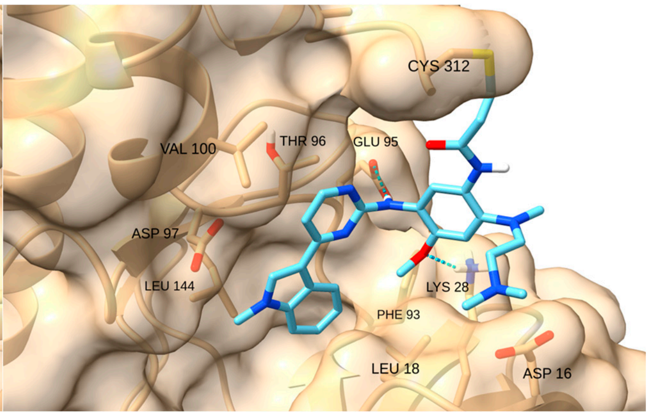

Osimertinib

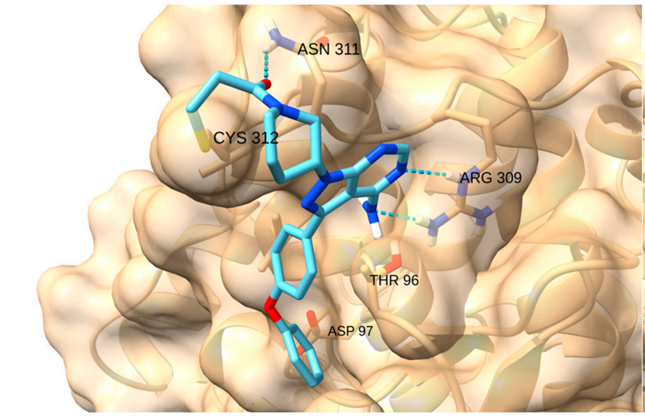

Ibrutinib

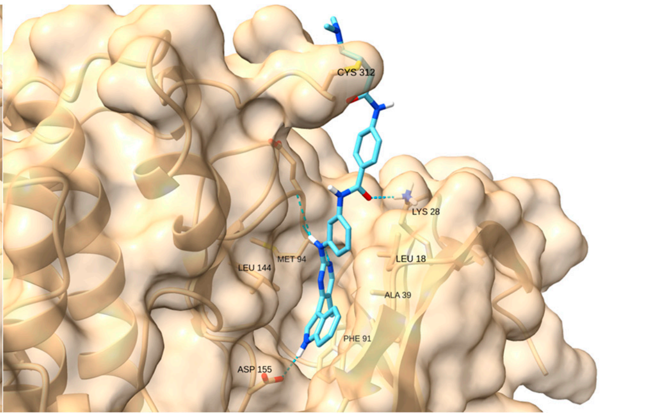

THZ1

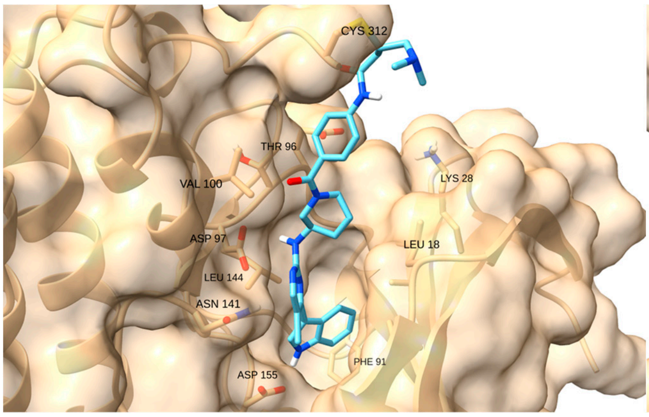

THZ531

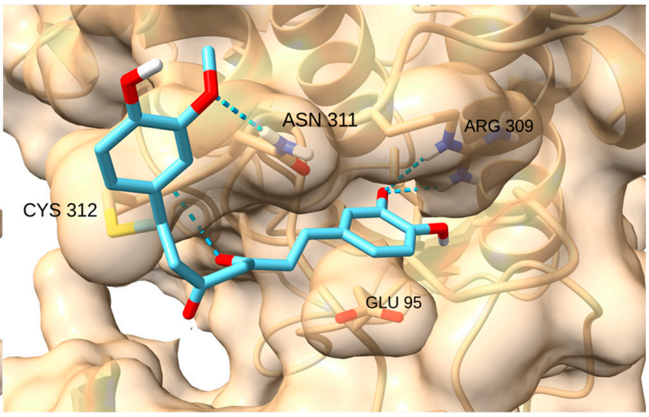

Curcumin

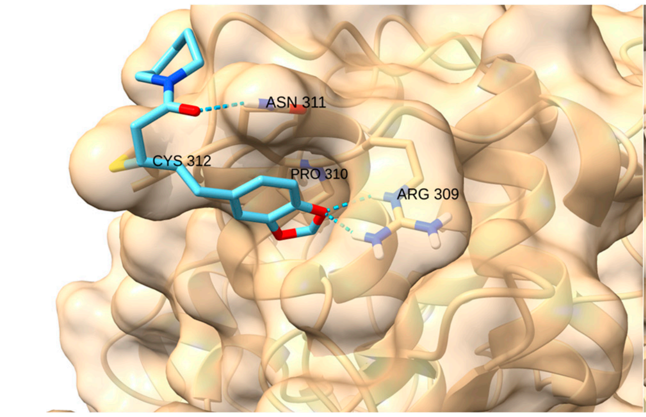

Piperine

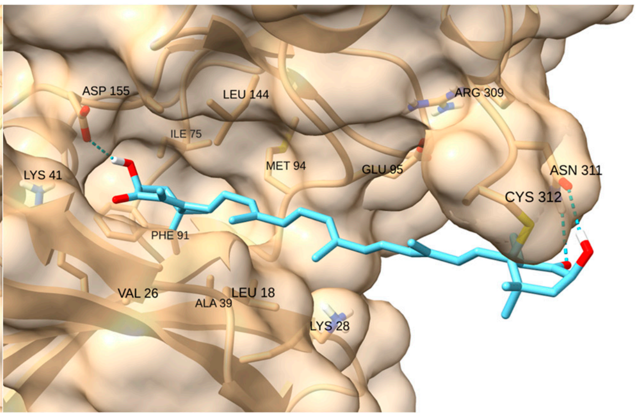

Astaxanthin

Figure S5. All docking poses for natural and reference covalent inhibitors in CDK7 with ligands Sunitinib, Osimertinib, Ibrutinib, THZ1, THZ531, Curcumin, Piperine, and Astaxanthin. Reactive cysteines are shown in yellow (Sγ).

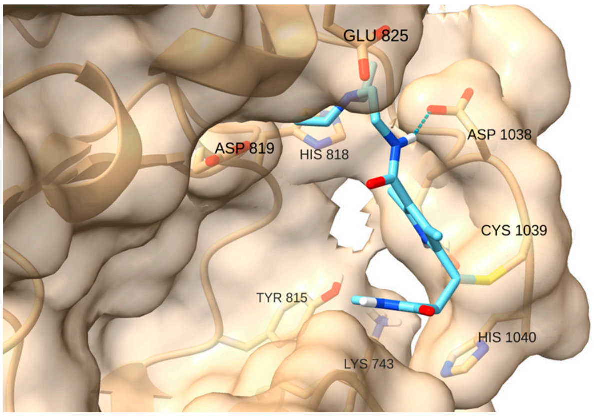

Sunitinib

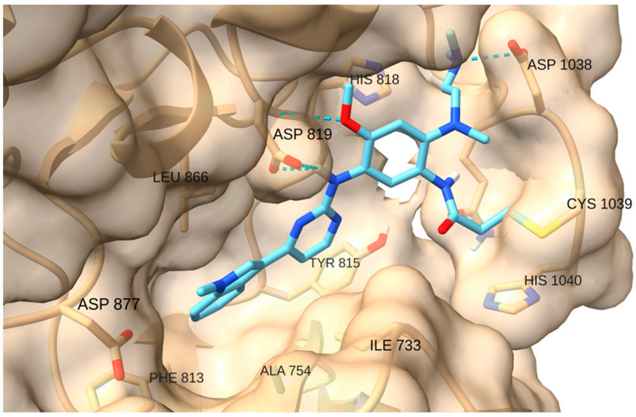

Osimertinib

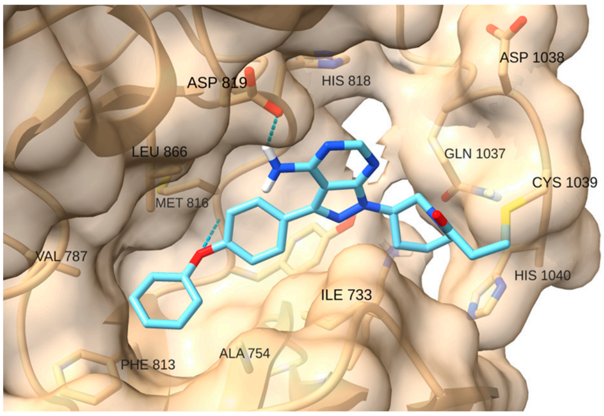

Ibrutinib

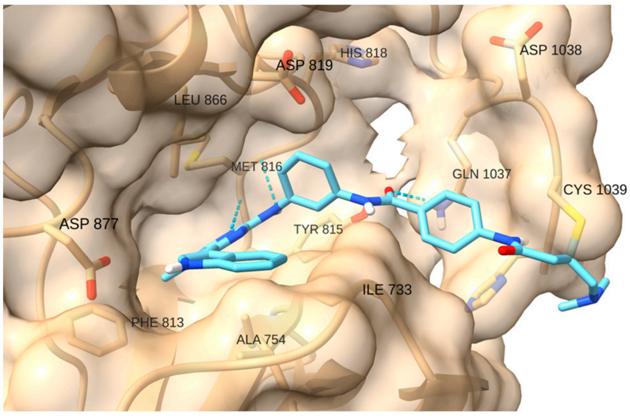

THZ1

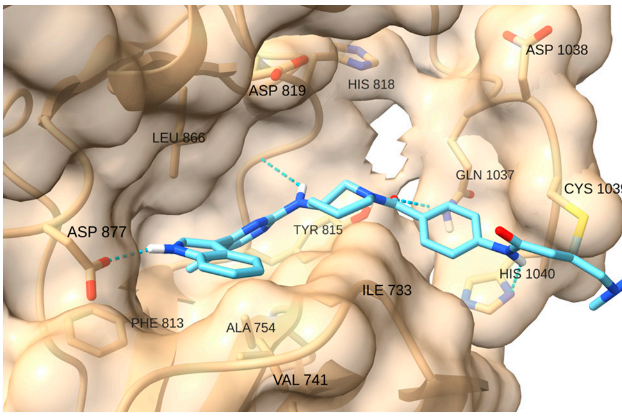

THZ531

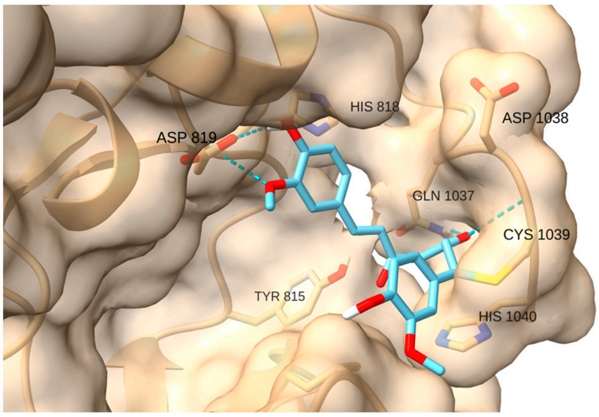

Curcumin

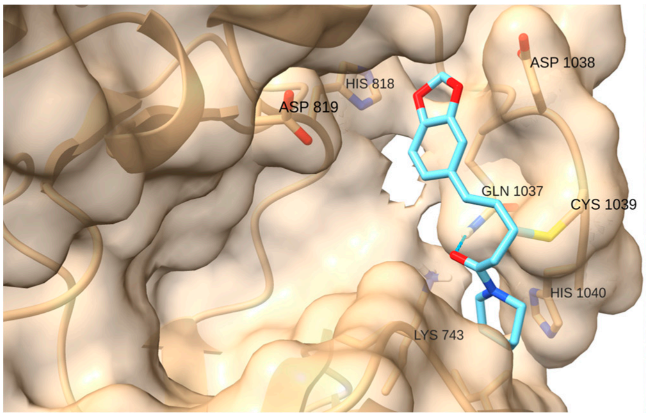

Piperine

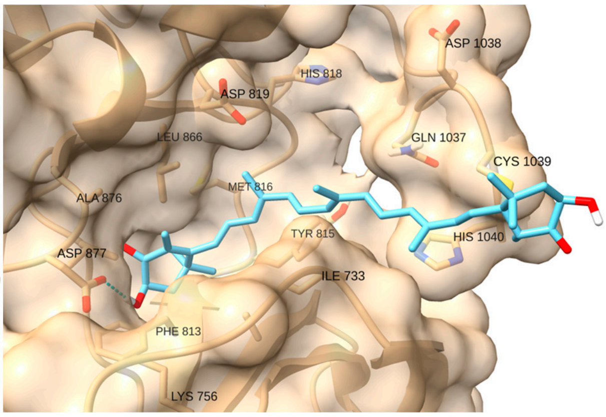

Astaxanthin

Figure S6. All docking poses for natural and reference covalent inhibitors in CDK12 with ligands Sunitinib, Osimertinib, Ibrutinib, THZ1, THZ531, Curcumin, Piperine, and Astaxanthin. Reactive cysteines are shown in yellow (Sγ).

Table S1: Docking energies (kcal/mol) from AutoDockFR for each ligand–receptor pair, reported for within-receptor ranking only (not for quantitative comparison). Docking was performed using AutoDockFR software suit version 1.0 rcl for Linux, incorporating flexible residues and covalent docking protocols. Positive values are in red.

Ligand	EGFR CYS797	VEGFR2 CYS919	PDGFR CYS814	BTK CYS481	CDK7 CYS312	CDK12 CYS1039
**Sunitinib**	-5.9	-2.4	-6.4	-5.6	-2.8	-6.2
**Osimertinib**	-7.6	-5.0	-7.8	-6.2	-5.7	-9.0
**Ibrutinib**	-9.1	-6.9	-8.4	-10.0	-5.6	-7.3
**Thz1**	-9.8	-5.7	-7.4	-6.6	-6.7	-8.9
**Thz531**	-6.6	-6.7	-7.6	-6.6	-6.8	-8.1
**Curcumin**	-6.7	-5.5	-6.6	-5.5	-4.0	-5.1
**Piperine**	-6.3	-6.9	-6.2	-6.6	-2.8	-4.4
**Astaxanthin**	-10.2	+7.0	-6.1	-9.1	-6.6	-7.1
